# Supplementary material for: Data quality in online human-subjects research: Comparisons between MTurk, Prolific, CloudResearch, Qualtrics, and SONA
Source: PLoS One. 2023 Mar 14;18(3):e0279720. doi: 10.1371/journal.pone.0279720 (PMC10013894; doi:10.1371/journal.pone.0279720)
Supplement: S1 Appendix — (DOCX) [file pone.0279720.s001.docx]

Supplemental Appendix 1

Data Quality in Human Subjects Research

Benjamin D. Douglas

Patrick J. Ewell

Markus Brauer

The primary purpose of our study was to examine how various data collection platforms differed with respect to the percentages of high-quality participants when randomly sampling from each platform. In the paragraphs below we describe results from the preliminary study used to identify which platforms to include in our main study. We also include two additional results concerning the representativeness of each sample and how data quality is affected by removing low-quality participants.

**Identifying Online Platforms**

Participants (*N* = 206) were researchers who volunteered to take the survey. Participants were contacted through the Society for Personality and Social Psychology’s email listserv. This listserv was selected because it reaches a broad range of disciplines including psychology, marketing, and political science to name a few. Participants included an undergraduate student (*n* = 1), lab managers and full-time research assistants (*n* = 2), graduate students (*n* = 68), postdoc researchers (*n* = 19), pre-tenure faculty (*n* = 46), post-tenure faculty (*n* = 62), and individuals with another position (*n* = 6).

Participants were asked to indicate the frequency with which they used various online survey platforms including Amazon’s Mechanical Turk (MTurk), CloudResearch’s MTurk Toolkit, CloudResearch’s Prime Panels, Dynata (Formally Survey Sampling International), Lucid, Prolific, Qualtrics’ Survey Platform, SurveyMonkey’s paid panels, an undergraduate student sample, or another survey platform. Frequency was assessed with open-response questions in which participants reported an integer representing the number of times they had used each platform over the previous three years. Participants were also asked to indicate their current occupation.

The five most used platforms were an undergraduate sample (*M* = 6.09, *SD* = 6.84), MTurk (*M* = 4.90, *SD* = 8.58), CloudResearch’s MTurk Toolkit (*M* = 4.01, *SD* = 8.51), Prolific (*M* = 3.21, *SD* = 5.52), and Qualtrics’ Survey Platform (*M* = 1.56, *SD* = 4.20). Following the most frequently used platforms were another (not listed) platform (*M* = 0.90, *SD* = 2.50), CloudResearch’s Prime Panels (*M* = 0.48, *SD* = 2.02), SurveyMonkey (*M* = 0.87, *SD* = 0.43), Lucid (*M* = 0.09, *SD* = 0.79), and Dynata (*M* = 0.03, *SD* = 0.22).

A post-hoc examination of articles published from 2020 – 2021 in Nature Human Behavior, the American Journal of Political Science, the Journal of Personality and Social Psychology, and Academy of Management’s journal confirmed that the five most frequently used platforms were MTurk (N = 259), Prolific (N = 67), CloudResearch (N = 37), Qualtrics (N = 18), and Undergraduate Panel (N = 12). These were followed by Dynata (N = 8), Lucid (N = 7), YouGov (N = 6), and Ariadna Research Panel (N = 5). These results support our findings that the five panels we evaluated are indeed the five panels most commonly used across disciplines.

**Representativeness**

A comparison of the demographic data revealed that Qualtrics participants were on average older and SONA participants (primarily college-aged students) were younger than participants on the other platforms (see SA Table 1). Participants on Qualtrics and SONA were also more likely to be female. Participants on Qualtrics were more likely to be White and conservative than participants on the other platforms. Participants on SONA were more likely to be from the top two income brackets (income > $100,000 per year) than any of the other platforms.

**SA Table 1**

***Demographic Information by Platform***

| **Measure** | **MTurk**  **(N = 500)** | **CloudResearch**  **(N = 505)** | **Prolific**  **(N = 496)** | **Qualtrics**  **(N = 575)** | **SONA**  **(N = 555)** | **National Sample** |
| --- | --- | --- | --- | --- | --- | --- |
| *Age*^1^ | 38.75 (11.53) | 41.99 (12.92) | 37.23 (14.01) | 64.34 (13.06) | 18.53 (1.27) | 38.50 |
| *Gender*^2^ |  |  |  |  |  |  |
| Female | 36.20% | 50.50% | 67.54% | 66.61% | 62.34% | 50.50% |
| Male | 63.40% | 48.32% | 30.44% | 32.70% | 29.01% | 47.20% |
| Another identity | 0.00% | 0.40% | 1.41% | 0.35% | 0.36% | - |
| Prefer not to say | 0.40% | 0.79% | 0.60% | 0.17% | 0.36% | 0.60% |
| *Identify as Transgender*^2^ |  |  |  |  |  |  |
| Yes | 7.00% | 0.99% | 2.42% | 0.87% | 0.72% | 1.70% |
| No | 92.00% | 98.22% | 96.37% | 98.43% | 90.99% | 97.70% |
| I am unsure | 0.00% | 0.20% | 0.20% | 0.17% | 0.36% | - |
| Prefer not to say | 0.80% | 0.59% | 1.01% | 0.52% | 0.00% | 0.60% |
| *Ethnicity*^3^ |  |  |  |  |  |  |
| American Indian or Alaskan Native | 1.20% | 0.99% | 2.02% | 0.70% | 0.72% | 1.30% |
| Asian or Asian American | 4.00% | 9.31% | 12.90% | 0.70% | 21.44% | 5.90% |
| Black or African American | 15.00% | 7.72% | 9.88% | 1.74% | 3.78% | 13.40% |
| Latino, Hispanic, Chicano, or Puerto Rican | 5.80% | 4.95% | 8.67% | 2.43% | 6.49% | 18.50% |
| Middle Eastern, Arab American, or North African | 0.40% | 0.79% | 0.60% | 0.35% | 1.80% | - |
| Native Hawaiian or Pacific Islander | 0.00% | 0.59% | 0.00% | 0.00% | 0.36% | 0.20% |
| White or European | 75.40% | 79.01% | 72.38% | 94.96% | 67.93% | 76.30% |
| Another Identity | 0.60% | 1.19% | 0.60% | 0.35% | 0.36% | - |
| Prefer not to say | 1.00% | 0.99% | 0.40% | 0.52% | 0.36% | - |
| *Sexual Orientation*^2^ |  |  |  |  |  |  |
| Straight or Heterosexual | 82.60% | 89.70% | 79.23% | 92.70% | 79.28% | 88.30% |
| Gay or Homosexual | 1.80% | 2.97% | 3.23% | 2.43% | 1.98% | 3.30% |
| Bisexual | 15% | 4.55% | 13.71% | 2.78% | 7.93% | 4.40% |
| Another identity | 0.20% | 1.19% | 2.02% | 0.52% | 0.54% | 1.90% |
| Prefer not to say | 0.40% | 1.58% | 1.81% | 1.57% | 2.16% | - |
| *Income*^4^ |  |  |  |  |  |  |
| Less than $10,000 | 2.00% | 2.77% | 6.25% | 3.48% | 1.44% | 5.80% |
| $10,000 - $19,999 | 4.60% | 5.74% | 7.26% | 10.78% | 0.90% | 32.60% |
| $20,000 - $29,999 | 9.00% | 7.92% | 9.07% | 12.17% | 2.16% |  |
| $30,000 - $39,999 | 11.00% | 10.30% | 10.48% | 11.65% | 3.96% |  |
| $40,000 - $49,999 | 15.40% | 10.30% | 11.69% | 9.39% | 4.68% |  |
| $50,000 - $59,999 | 19.00% | 12.87% | 9.07% | 9.74% | 5.23% | 30.20% |
| $60,000 - $69,999 | 7.60% | 8.71% | 8.27% | 7.83% | 4.68% |  |
| $70,000 - $79,999 | 8.80% | 9.50% | 9.27% | 6.96% | 6.13% |  |
| $80,000 - $89,999 | 7.00% | 5.74% | 4.44% | 5.39% | 3.42% |  |
| $90,000 - $99,999 | 7.40% | 7.13% | 5.44% | 4.35% | 4.68% |  |
| $100,000 - $149,999 | 6.40% | 12.48% | 10.48% | 12.52% | 18.38% | 15.70% |
| More than $150,000 | 1.80% | 5.94% | 7.86% | 5.74% | 35.68% | 15.70% |
| *Highest level of education completed*^5^ |  |  |  |  |  |  |
| Less than high school education | 0.20% | 0.59% | 0.40% | 0.70% | 0.18% | - |
| High school graduate | 5.00% | 6.93% | 12.90% | 22.26% | 48.29% | 26.90% |
| Some college | 7.00% | 17.43% | 24.40% | 21.04% | 40.18% | 20.00% |
| 2-year degree | 5.40% | 10.30% | 10.69% | 13.57% | 0.72% | 8.60% |
| 4-year degree | 60.00% | 46.53% | 36.29% | 25.91% | 2.16% | 20.30% |
| Master’s degree | 21.80% | 14.85% | 11.09% | 13.57% | 0.18% | 12.80% |
| Doctorate or professional degree | 0.60% | 3.37% | 4.03% | 2.96% | 0.18% |  |
| *Political Affiliation (ANES)*^6^ |  |  |  |  |  |  |
| Strong Republican | 21.60% | 12.28% | 6.85% | 24.00% | 5.23% | 21.03% |
| Weak Republican | 7.60% | 12.67% | 7.66% | 12.52% | 13.51% | 10.62% |
| Independent Republican | 2.20% | 8.12% | 5.44% | 6.43% | 7.39% | 10.48% |
| Independent Independent | 3.60% | 10.30% | 11.69% | 13.74% | 13.51% | 11.83% |
| Independent Democrat | 3.40% | 10.50% | 13.71% | 6.61% | 9.73% | 11.43% |
| Weak Democrat | 15.60% | 20.00% | 19.35% | 13.57% | 22.70% | 11.54% |
| Strong Democrat | 46.00% | 25.94% | 35.28% | 23.13% | 19.64% | 23.19% |
| *Party Affiliation*^7^ |  |  |  |  |  |  |
| Republican Party | 28.80% | 32.87% | 19.35% | 46.09% | 22.88% | 26.00% |
| Libertarian Party | 2.60% | 6.34% | 6.45% | 2.78% | 7.57% | - |
| Democratic Party | 67.60% | 56.24% | 62.90% | 47.83% | 55.14% | 42.00% |
| Green Party | 1.00% | 3.56% | 11.09 | 3.30% | 5.95% | - |
| Independent | - | - | - | - | - | 29.00% |

*Note*. SA Table 1 includes mean and standard deviation for age by platform. For all other demographic measures, we report the percentage of participants who selected each response by platform. Participants could select more than one option for ethnicity. Some percentages may not add to 100% because of missing responses. In the final column we present national demographic information. Some categories do not directly correspond with one another (e.g., national information is only available for sex, not gender). The following sources were used to provide the comparison statistic for each demographic measure: United States Census Bureau^1^ [1], Anderson et al. ^2^ [2], United States Census Bureau^3^ [3], United States Census Bureau^4^ [4], United States Census Bureau^5^ [5], American National Election Studies^6^ [6], Gallup^7^ [7].

To evaluate how the demographic data from each of the five platforms compared with national demographic data, we used data collected by the United States Census Bureau, Gallop, and the American National Election Studies for the equivalent demographic measures collected in our study. National demographic data is presented in the final column of SA Table 1. Participants on Qualtrics were older and participants on SONA were younger than the US national average. Likewise, women were overrepresented on Prolific, Qualtrics, and SONA, while they were underrepresented on MTurk. Qualtrics also had an overrepresentation of White participants while SONA had an overrepresentation of Asian or Asian American participants. Participants on SONA (college students) were also more likely to be in the top income brackets than the national average, likely a result of the students reporting their parent’s income rather than their own. With respect to education, Qualtrics participants most closely reflected the US population while MTurk least closely reflected the US population. Democrats were overrepresented on MTurk, CloudResearch, Prolific and SONA while Republicans were overrepresented on Qualtrics and independents were underrepresented on all platforms. Overall, while none of the samples were perfectly representative, CloudResearch participants were the most similar to the US population, generally reflecting the national samples in all categories, except for education and (to a lesser extent) political affiliation.

While these demographic results provide insight as to how well each sample represents the US population, it is important to note that without repeatedly testing each of these platforms one cannot conclude that a given platform is definitively more representative of the US population than another platform. Furthermore, researchers using online data collection platforms have the option to use or not use quotas when recruiting participants. Quotas allow the researcher to specify the demographic characteristics of their sample. For example, researchers could use quotas to allow no more than 500 women out of 1000 total participants to take a survey. This hypothetical quota would make the distribution of women in the survey similar to the gender distribution in the US population. In the present study we opted to not use quotas to “see what you get” when posting a study to each of the data collection platforms. Researchers considering using any of these platforms should be aware that they can specify the demographic distributions of the participants in their study, however doing so will likely increase the cost of conducting the study.

**Removal of Low-Quality Respondents**

Whereas the purpose of this study was to better understand which platforms produce the highest quality data, a secondary question arises: once examining only high quality participants, do the differences between platforms on the indirect measures of data quality change? Numerous studies have found that low quality respondents affect a study’s results [8-10]. Thus, it is reasonable to assume that we might find the effect with our own data.

To determine how our results were affected by the presence of low quality respondents on each platform, in SA Table 2 we re-present a portion of the continuous outcome measures without the inclusion of the low-quality respondents. As was the case for the original version of the table, we ran 11 inferential tests per outcome measure, 10 tests that compared each platform to each of the other platforms and 1 overall 4-df test examining the null hypothesis that there were no group differences. We used dummy codes to do pairwise comparisons between platforms. We created a set of four dummy codes with MTurk as the reference group and tested their statistical significance without any kind of adjustment. We again did post-hoc pairwise comparisons between Prolific, CloudResearch, Qualtrics, and SONA using different sets of dummy codes and applying a Holm-Bonferroni adjustment to determine statistical significance (i.e., to account for the fact that we already “used up” all available degrees of freedom for the test of our a priori hypotheses) [12].

**SA Table 2**

***Descriptive Statistics for the Continuous Outcome Measures Directly or Indirectly Related to Data Quality, Broken Down by Platform after the Removal of Low Quality Respondents***

| **Measure** | **MTurk**  **(N = 132)** | **CloudResearch**  **(N = 313)** | **Prolific**  **(N = 337)** | **Qualtrics**  **(N = 306)** | **SONA**  **(N = 293)** |
| --- | --- | --- | --- | --- | --- |
| Personality Inventory (mean scores) |  |  |  |  |  |
| Neuroticism* | 2.35^a^ (0.85) | 2.33^a^ (1.01) | 2.82^b^ (0.97) | 2.34^a^ (0.80) | 2.70^b^ (0.80) |
| Extroversion* | 3.06^a^ (0.88) | 2.96^a^ (0.89) | 2.90^a^ (0.91) | 2.99^a^ (0.76) | 3.50^b^ (0.77) |
| Openness* | 3.62^a^ (0.72) | 3.78^b^ (0.72) | 3.96^c^ (0.68) | 3.36^d^ (0.70) | 3.71^ab^ (0.57) |
| Agreeableness* | 3.81^a^ (0.67) | 3.93^ab^ (0.66) | 3.78^a^ (0.59) | 3.99^b^ (0.57) | 3.90^ab^ (0.53) |
| Conscientiousness* | 3.94^a^ (0.73) | 4.05^a^ (0.70) | 3.63^b^ (0.79) | 3.97^a^ (0.63) | 3.66^b^ (0.63) |
| Personality Inventory (Cronbach’s Alphas) |  |  |  |  |  |
| Neuroticism (IPIP = .86) | 0.87 | 0.94 | 0.91 | 0.90 | 0.88 |
| Extraversion (IPIP = .86) | 0.88 | 0.91 | 0.90 | 0.89 | 0.89 |
| Openness (IPIP = .82) | 0.78 | 0.83 | 0.81 | 0.80 | 0.72 |
| Agreeableness (IPIP = .77) | 0.81 | 0.86 | 0.79 | 0.85 | 0.78 |
| Conscientiousness (IPIP = .81) | 0.87 | 0.89 | 0.89 | 0.88 | 0.82 |
| Belief in Conspiracy Theories* | 2.58^a^ | 2.37^bc^ | 2.54^ab^ | 2.32^c^ | 2.42^abc^ |
| Correlation Between T1 and T2 Response to the Same Question* | 0.82^a^ | 0.92^b^ | 0.87^ab^ | 0.88^ab^ | 0.68^c^ |

*Note*. The reported values are mean values for each platform. Standard deviations are reported in parentheses. Means with different superscripts a-d are significantly different at the *p* < .05 threshold. A * indicates that the overall 4-df test was statistically significant. The IPIP values represent Cronbach’s Alphas values reported by the International Personality Inventory Pool and come from an existing sample of *N* = 856 participants [13].

As was the case for previous studies, our results changed after removing the low quality respondents, particularly for the MTurk sample. When including all participants, the MTurk sample has alpha values of 0.70, 0.67, and 0.65 for the neuroticism, extraversion, and openness subscales respectively. After the removal of the low quality respondents the alpha values for these subscales were 0.87, 0.88, and 0.78. After the removal of low quality participants, mean scores for each of the personality subscales for the MTurk sample became more similar to scores on other platforms (particularly CloudResearch). While MTurk and CloudResearch originally had significantly different mean scores for all five personality subscales, after the removal of low quality participants these two platforms differed only on the openness subscale. Likewise, the mean score for belief in conspiracy theories dropped from 3.26 on MTurk to 2.58 after removing low quality respondents, making it more similar to other platforms. While having particularly high scores on a personality measure or belief in conspiracies does not mean a platform has inherently lower quality respondents, the fact that these scores change after removing low quality respondents does indicate that one’s results can be affected by the inclusion of low-quality respondents. As such, researchers conducting online studies would be advised to use platforms that offer a greater proportion of high quality participants. Furthermore, researchers would be advised to adopt a (preregistered) procedure for identifying and removing low quality respondents from their data.

**References**

1. United Staes Census Bureau. 2019. Age and Sex [dataset]. Available from: <https://data.census.gov/cedsci/table?q=United%20States&g=0100000US&tid=ACSST1Y2019.S1501https://data.census.gov/cedsci/table?q=age&tid=ACSST1Y2019.S0101>
2. Anderson L, File F, Marshall J, McElrath K, Sherer Z. New Household Pulse Survey Data Reveals Differences between LGBT and Non-LGBT Respondents During COVID-19 Pandemic. 2021 Nov 4. Available from: https://www.census.gov/library/stories/2021/11/census-bureau-survey-explores-sexual-orientation-and-gender-identity.html
3. United States Census Bureau. 2020. Hispanic or Latino, And Not Hispanic or Latino By Race [dataset]. Available From: <https://data.census.gov/cedsci/table?q=ethnicity&g=0100000US>
4. United States Census Bureau. 2019. Educational Attainment [dataset]. Available From: <https://data.census.gov/cedsci/table?q=United%20States&g=0100000US&tid=ACSST1Y2019.S1501>
5. United States Census Bureau. 2019. Income in the Past 12 Months (In 2019 Inflation-Adjusted Dollars) [dataset]. Available From: <https://data.census.gov/cedsci/table?q=United%20States&g=0100000US&tid=ACSST1Y2019.S1901>
6. The American National Election Studies (ANES). 2020. Party Identification 7-Point Scale 1952-2020 [dataset]. Available from: https://electionstudies.org/resources/anes-guide/top-tables/?id=21
7. Gallup. Party Affiliation [dataset]. Available from <https://news.gallup.com/poll/15370/party-affiliation.aspx>
8. Credé M. Random responding as a threat to the validity of effect size estimates in correlational research. Educ Psychol Meas. 2010; 70: 596-612.
9. Huang JL, Liu M, Bowling NA. Insufficient effort responding: examining an insidious confound in survey data. J Appl Psychol. 2015; 100: 828.
10. Huang JL, DeSimone JA. Insufficient effort responding as a potential confound between survey measures and objective tests. J Bus and Psychol. 2021; 36: 807-828.
11. Holm S. A simple sequentially rejective multiple test procedure. Scand Stat Theory Appl. 1979; 65-70.
12. Goldberg LR, Johnson JA, Eber HW, Hogan R, Ashton MC, Cloninger CR, Gough HG. The international personality item pool and the future of public-domain personality measures. J Res Pers. 2006; 40: 84-96.
