# Supplementary material for: Data quality in online human-subjects research: Comparisons between MTurk, Prolific, CloudResearch, Qualtrics, and SONA
Source: PLoS One. 2023 Mar 14;18(3):e0279720. doi: 10.1371/journal.pone.0279720 (PMC10013894; doi:10.1371/journal.pone.0279720)
Supplement: S1 Data — (DOCX) [file pone.0279720.s002.docx]

Data quality in online human-subjects research:

Comparisons between MTurk, Prolific, CloudResearch, Qualtrics, and SONA

Benjamin D. Douglas^1^, Patrick J. Ewell^2^, Markus Brauer^1*^

^1^ Department of Psychology, University of Wisconsin–Madison.

^2^ Department of Psychology, Kenyon College.

* Corresponding author

Email: markus.brauer@wisc.edu

**Abstract**

With the proliferation of online data collection in human-subjects research, concerns have been raised over the presence of inattentive survey participants and non-human respondents (bots). We compared the quality of the data collected through five commonly used platforms. Data quality was indicated by the percentage of participants who meaningfully respond to the researcher’s question (high quality) versus those who only contribute noise (low quality). We found that compared to MTurk, Qualtrics, or an undergraduate student sample (i.e., SONA), participants on Prolific and CloudResearch were more likely to pass various attention checks, provide meaningful answers, follow instructions, remember previously presented information, have a unique IP address and geolocation, and work slowly enough to be able to read all the items. We divided the samples into high- and low-quality respondents and computed the cost we paid per high-quality respondent. Prolific ($1.90) and CloudResearch ($2.00) were cheaper than MTurk ($4.36) and Qualtrics ($8.17). SONA cost $0.00, yet took the longest to collect the data.

**Introduction**

As online data collection for human subjects through platforms like Amazon’s Mechanical Turk (MTurk) becomes increasingly common [1], so too have concerns over the quality of these data. Do participants on these platforms provide meaningful responses? A recent study found that data quality from respondents on MTurk has decreased since 2015 [2]. That is, the number of incoherent responses to open ended questions, inconsistent responses to the same questions, responses in which participants report experiencing something impossible or highly improbable, and patterns of responses indicating inattentive survey taking have increased. Such patterns of responding are of particular concern as researchers have found that low-quality respondents can confound established correlations between variables, either strengthening them [3-5] or, in some instances, changing the direction of the correlation [5]. While there is evidence that data screening procedures can improve data quality, these results are mixed [6]. Furthermore, even with the ability to clean data post-hoc, paying for participants can be expensive for researchers. Thus, it is important for researchers to understand their options with respect to online data collection and the quality of the data that each online platform provides. We investigated which of the most frequently used online data collection platforms produce the highest data quality.

**Defining High-Quality Data**

To be able to compare the data quality of various platforms, it is important to first clarify what we mean when we describe a participant’s response as being high or low quality. Researchers have identified various categories of low-quality responding: inattentive respondents (those who hastily take a survey or do not follow the study’s explicit directions), dishonest respondents (those who deliberately provide false information), respondents who fail to comprehend a study’s directions, or unreliable respondents (those who provide different responses over time) [7]. Please note that our use of the term “data quality” does not concern whether a sample generalizes to a larger population. We only address data quality with respect to the percentage of participants who meaningfully respond to the researcher’s question (high quality) versus those who only contribute noise (low quality).^[[1]](#footnote-1)^

**Data Quality for Online Data Collection**

In general, researchers examining data quality from online survey platforms have consistently found large proportions of low-quality responses [8-10]. Likewise, the Pew Research Center has noted as much as 4% of responses to online polls are from low-quality participants [11]. Given that some responses will inevitably be low-quality when conducting online research, researchers using these platforms benefit from knowing which platforms offer the highest quality data.

Previous studies concerning data quality have mainly focused on MTurk and have found mixed results. Initially, the studies found that alpha values for personality scales remained within one hundredth of alphas based on in-person data collection, even when MTurk participants were paid as little as $0.01 [12]. Likewise, Roulin [13] noted several benefits to collecting data through MTurk instead of other commercial data collection platforms such as Qualtrics, including: high-quality data, the ability to collect data inexpensively, and a participant sample that better represents the general population. Other researchers observed that MTurk produces data of comparable quality to student samples and superior quality data to other forms of professional platforms like Qualtrics and Lightspeed [14]. In yet another study, Smith and colleagues [15] compared US and non-US based MTurk participants with a Qualtrics sample and found that while MTurk workers spent less time responding to the items, they correctly responded to the attention checks scattered through the survey. Taken together, these studies provided initial support for the idea that high-quality data can be obtained via MTurk.

High-quality data are not guaranteed on MTurk as has been observed in numerous studies, particularly those conducted more recently. In a study examining how payment might incentivize data quality in both US and India-based MTurk samples, Litman and colleagues [16] demonstrated that participants were motivated by compensation and provided higher quality data with higher pay. In comparison with both a community and campus pool of participants, MTurk participants self-reported providing lower quality responses and were more experienced taking surveys than either of the comparison samples [17]. In another study, participants were asked if they were colorblind followed by a series of colorblindness tests [18]. These colorblindness tests were designed so that some images would be visible for all participants, regardless of colorblindness or consisted of questions about types of colorblindness which do not exist (i.e., red-blue colorblindness). The authors determined that more than half of the participants misreported having colorblindness [18]. Yet another study using the HEXACO-96 personality measure found that upwards of 15% of participants gave noncompliant, or seemingly random, responses [6]. Also, findings of poor-quality data have become increasingly frequent as demonstrated over the course of a four-year period of testing the psychometric properties of the Big-5 Personality Inventory (i.e., Cronbach’s alphas and internal validity between scales) [2]. To summarize, concerns over MTurk data quality are increasingly warranted.

While there are many studies on the topic of data quality in MTurk samples, less research has examined alternative platforms. One such study examined the data quality of Prolific.ac, CrowdFlower, and a student participant pool in addition to MTurk [19]. The authors’ indicators of data quality included time spent taking the survey, the accuracy in responding to the attention checks, and the reliability of the established psychological measures. Participants were also asked about their frequency taking online studies. Results indicated that Prolific participants took the fewest number of studies. Additionally, while CrowdFlower participants failed the most attention checks, both CrowdFlower and Prolific participants had lower levels of dishonest behavior than the MTurk participants. Overall, Prolific was recommended as an alternative to MTurk [19]. While these results are promising for Prolific, more research is needed to establish Prolific as a definitively superior option over MTurk in terms of data quality.

Among the more frequently used alternatives to MTurk is CloudResearch (previously known as TurkPrime). CloudResearch features multiple methods for online data collection including the MTurk Toolkit (which interfaces directly with MTurk) and Prime Panels (which recruits participants independently from MTurk). One study comparing data quality between MTurk and CloudResearch’s Prime Panels found that once the initial attention checks were used to screen out participants, Prime Panels had a more diverse, less experienced sample and provided better data quality than MTurk. However, more Prime Panel participants failed the initial attention checks than MTurk participants [20]. In another study, researchers examined how easy it was to recruit a difficult-to-reach population (namely individuals interested in participating in a smoking-cessation study) on various platforms [21]. The authors recruited participants through CloudResearch’s MTurk Toolkit, Qualtrics, Soapbox Sample, and Reddit. More participants from the desired population were recruited from Soapbox Sample than any other platform, but the participants recruited through CloudResearch provided the highest quality data. Given the small number of studies and the somewhat inconsistent results, we cannot yet draw conclusions about how CloudResearch’s data quality compares with that of other data collection platforms.

Recent events related to the COVID-19 pandemic have led to changes on data collection platforms which likely affect the quality of data obtained through these platforms. Due to the financial crisis starting in 2020, the demographics of MTurk workers are now more representative of the overall US population—more Republican, male, and non-White [22]. However, these new participants also appear to be less experienced with online surveys and provide less thoughtful responses. Worse data quality has also been found following the COVID-19 pandemic on Lucid [23], another data collection platform which had previously been recommended as a viable alternative to MTurk [24]. These results suggest that not only is it important to understand data quality for online platforms generally, but also we are currently undergoing a period of change on these platforms and should take particular note of data quality in the wake of the COVID-19 pandemic.

One final reason it is important to examine data quality is to gain an independent perspective on data quality not influenced by researchers affiliated with the data collection platforms. A recent controversy arose between researchers affiliated with Prolific and CloudResearch when individuals affiliated with Prolific (including the company’s CEO) published findings stating that Prolific offered the highest quality data among a series of data collection platforms including CloudResearch [7]. The CloudResearch team offered a rebuttal after identifying that the Prolific team had disabled many of CloudResearch’s data quality filters [25]. The Prolific researchers subsequently republished their findings with a second study which used CloudResearch’s filters [7]. This exchange between Prolific and CloudResearch highlights the importance of disinterested science. As such, the present research expands upon these existing findings by testing data quality from the perspective of researchers unaffiliated with any data collection platform.

As online data collection becomes increasingly common, researchers will benefit from knowing which platforms produce the highest quality data. However, existing studies examining data quality have been largely restricted to MTurk. Likewise, these studies do not account for recent changes in data quality, such as those caused by the COVID-19 pandemic and the associated financial crisis. We therefore decided to do a rigorous comparison of five of the most commonly used online data collection platforms.

**Methodological Approaches for Testing Data Quality**

Researchers have used a variety of indicators to assess data quality. Among the most common indicators of data quality are attention checks. Attention checks allow researchers to examine if a participant provides meaningful responses at different stages throughout the survey. Thus, attention checks are particularly useful in longer surveys as previous studies have shown that the longer a survey, the less careful participants become [26]. Although frequently used, the use of attention checks is not without concern. While Hauser et al. [27] found that instructional manipulation checks (a particular type of attention check) did not alter the observed effects of their study, Hauser et al. [28] subsequently discovered that researchers alter the psychological experience of taking a survey by including attention checks. As such, these authors recommend testing any attention checks prior to their use in a survey.

Likewise, researchers have found that some instances of non-compliant responding do not indicate inattentive responding. When presented with attention checks like “All my friends are aliens,” and asked to respond, some participants gave thoughtful responses such as “What does that even mean, we’re all aliens if there’s other life out there” [29]. While this is not the response the researchers likely were looking for, it does not indicate that the participants were inattentive. More than anything, this example highlights the importance of developing straightforward questions and attention checks that will not confuse participants. Additionally, attention checks do not catch all low-quality respondents. Barends and de Vries [6] found that while 15% of respondents gave incoherent responses to the survey, roughly 2% of the total sample nonetheless passed the instructional attention checks. While none of these findings suggest that one should abandon attention checks altogether, it is important to acknowledge the shortcomings of attention checks and use them in junction with other measures when testing for data quality.

Another indicator of data quality is the amount of time participants spend answering the survey questions. Researchers examining online data collection have established that speedy responses are associated with worse data quality [30-32]. More recently, Wood et al. [33] compared the number of seconds spent on each survey item with other indicators of data quality and established that when less than 1 second was spent per item, data quality dropped. The use of a 1 second per item speed cutoff has been subsequently used as an indicator of low-quality data [20; 34].

One other common data quality indicator is derived from the psychometric properties of existing scales, such as personality inventories. For example, in testing data quality on MTurk, Rouse [35] used the openness subscale of a Big-5 Personality Inventory measure and compared the alpha value for the MTurk sample with the alpha value found by Goldberg [36] who used a large-scale national sample. Even when participants who failed the attention checks were removed from the analyses, the alpha value for the MTurk sample was significantly lower than the one reported by Goldberg. Other personality measures have been used in many data quality studies [e.g., 2, 6, 16]. Among the most commonly used personality inventories is Costa and McCrae's [37] NEO-PI-R Domains obtained through the International Personality Item Pool (IPIP) [38] which includes 5 positive-keyed and 5 negative-keyed items for each trait of the Big-5. This inventory allows researchers to test for expected alpha values for each trait and is an established indicator of data quality [e.g., 20]. The NEO-PI-R Domains are particularly effective as they use the same number of positively and negatively keyed items, which is recommended when using alpha values to assess data quality [39]. As such, it is possible that the results from past studies that use scales with an unbalanced number of positively and negatively keyed items [e.g., 12] may be less accurate than studies which use balanced measures like the NEO-PI-R. Researchers conducting studies about data quality should be mindful of their scale items if they intend to use alpha values as an indicator of data quality.

Yet another indicator of data quality is suspicious responses to open-ended questions. For example, when given an open response box to report thoughts or ask questions at the end of the survey, responses written in all caps, one-word responses seemingly unrelated to the prompt, restatements of parts of the question, or nonsensical phrases have all been used as indicators of low-quality responses [2, 11].

Because there is no single best indicator of data quality, almost all studies on data quality use several methods. Using multiple approaches is ideal because the shortcomings of one data quality indicator are compensated by the strengths of other indicators. Given the advantages of combining data quality indicators, we decided to employ multiple methods such that we can accurately determine the quality of the data provided by a given platform.

**The Present Study**

The purpose of the present study was to assess the data quality of online data collection platforms. To this end, we examined the data quality on five frequently used platforms and determined which platform offers the best price per high-quality respondent.

**Method**

**Participants**

We recruited adult participants (*N* = 2729) from the United States through MTurk (*n*= 500), CloudResearch (*n* = 505), Prolific (*n* = 496), Qualtrics (*n* = 575), and an undergraduate student sample overseen by our department, SONA (*n* = 653). Because participants self-selected into each of the five data collection platforms, assignment to the five groups was not random. For all paid platforms (i.e., MTurk, CloudResearch, Prolific, and Qualtrics) only participants who submitted the survey received payment. As such, the attrition rate from these platforms was 0%. SONA participants had a higher attrition rate with 17.92% of participants (117 participants) providing only a partial response. We removed participants who progressed through less than 30% of the survey. A total of 555 participants recruited through SONA were included in our analyses. See Table 1 for a demographic breakdown of each of the five samples.

**Table 1. Demographic Information by Platform**

| **Measure** | **MTurk**  **(N = 500)** | **CloudResearch**  **(N = 505)** | **Prolific**  **(N = 496)** | **Qualtrics**  **(N = 575)** | **SONA**  **(N = 555)** |
| --- | --- | --- | --- | --- | --- |
| *Age* | 38.75 (11.53) | 41.99 (12.92) | 37.23 (14.01) | 64.34 (13.06) | 18.53 (1.27) |
| *Gender* |  |  |  |  |  |
| Female | 36.20% | 50.50% | 67.54% | 66.61% | 62.34% |
| Male | 63.40% | 48.32% | 30.44% | 32.70% | 29.01% |
| Another identity | 0.00% | 0.40% | 1.41% | 0.35% | 0.36% |
| Prefer not to say | 0.40% | 0.79% | 0.60% | 0.17% | 0.36% |
| No response | 0.00% | 0.00% | 0.00% | 0.17% | 7.93% |
| *Identify as Transgender* |  |  |  |  |  |
| Yes | 7.00% | 0.99% | 2.42% | 0.87% | 0.72% |
| No | 92.00% | 98.22% | 96.37% | 98.43% | 90.99% |
| I am unsure | 0.00% | 0.20% | 0.20% | 0.17% | 0.36% |
| Prefer not to say | 0.80% | 0.59% | 1.01% | 0.52% | 0.00% |
| No response | 0.20% | 0.00% | 0.00% | 0.00% | 7.93% |
| *Ethnicity* |  |  |  |  |  |
| American Indian or Alaskan Native | 1.20% | 0.99% | 2.02% | 0.70% | 0.72% |
| Asian or Asian American | 4.00% | 9.31% | 12.90% | 0.70% | 21.44% |
| Black or African American | 15.00% | 7.72% | 9.88% | 1.74% | 3.78% |
| Latino, Hispanic, Chicano, or Puerto Rican | 5.80% | 4.95% | 8.67% | 2.43% | 6.49% |
| Middle Eastern, Arab American, or North African | 0.40% | 0.79% | 0.60% | 0.35% | 1.80% |
| Native Hawaiian or Pacific Islander | 0.00% | 0.59% | 0.00% | 0.00% | 0.36% |
| White or European | 75.40% | 79.01% | 72.38% | 94.96% | 67.93% |
| Another Identity | 0.60% | 1.19% | 0.60% | 0.35% | 0.36% |
| Prefer not to say | 1.00% | 0.99% | 0.40% | 0.52% | 0.36% |
| No response | 0.00% | 0.20% | 0.00% | 0.00% | 8.11% |
| *Sexual Orientation* |  |  |  |  |  |
| Straight or Heterosexual | 82.60% | 89.70% | 79.23% | 92.70% | 79.28% |
| Gay or Homosexual | 1.80% | 2.97% | 3.23% | 2.43% | 1.98% |
| Bisexual | 15% | 4.55% | 13.71% | 2.78% | 7.93% |
| Another identity | 0.20% | 1.19% | 2.02% | 0.52% | 0.54% |
| Prefer not to say | 0.40% | 1.58% | 1.81% | 1.57% | 2.16% |
| No response | 0.00% | 0.00% | 0.00% | 0.00% | 8.11% |
| *Family Income* |  |  |  |  |  |
| Less than $10,000 | 2.00% | 2.77% | 6.25% | 3.48% | 1.44% |
| $10,000 - $19,999 | 4.60% | 5.74% | 7.26% | 10.78% | 0.90% |
| $20,000 - $29,999 | 9.00% | 7.92% | 9.07% | 12.17% | 2.16% |
| $30,000 - $39,999 | 11.00% | 10.30% | 10.48% | 11.65% | 3.96% |
| $40,000 - $49,999 | 15.40% | 10.30% | 11.69% | 9.39% | 4.68% |
| $50,000 - $59,999 | 19.00% | 12.87% | 9.07% | 9.74% | 5.23% |
| $60,000 - $69,999 | 7.60% | 8.71% | 8.27% | 7.83% | 4.68% |
| $70,000 - $79,999 | 8.80% | 9.50% | 9.27% | 6.96% | 6.13% |
| $80,000 - $89,999 | 7.00% | 5.74% | 4.44% | 5.39% | 3.42% |
| $90,000 - $99,999 | 7.40% | 7.13% | 5.44% | 4.35% | 4.68% |
| $100,000 - $149,999 | 6.40% | 12.48% | 10.48% | 12.52% | 18.38% |
| More than $150,000 | 1.80% | 5.94% | 7.86% | 5.74% | 35.68% |
| No response | 0.00% | 0.59% | 0.40% | 0.00% | 8.65% |
| *Highest level of education completed* |  |  |  |  |  |
| Less than high school education | 0.20% | 0.59% | 0.40% | 0.70% | 0.18% |
| High school graduate | 5.00% | 6.93% | 12.90% | 22.26% | 48.29% |
| Some college | 7.00% | 17.43% | 24.40% | 21.04% | 40.18% |
| 2-year degree | 5.40% | 10.30% | 10.69% | 13.57% | 0.72% |
| 4-year degree | 60.00% | 46.53% | 36.29% | 25.91% | 2.16% |
| Master’s degree | 21.80% | 14.85% | 11.09% | 13.57% | 0.18% |
| Doctorate or professional degree | 0.60% | 3.37% | 4.03% | 2.96% | 0.18% |
| No response | 0.00% | 0.00% | 0.20% | 0.00% | 8.11% |
| *Political Affiliation (ANES)* |  |  |  |  |  |
| Strong Republican | 21.60% | 12.28% | 6.85% | 24.00% | 5.23% |
| Weak Republican | 7.60% | 12.67% | 7.66% | 12.52% | 13.51% |
| Independent Republican | 2.20% | 8.12% | 5.44% | 6.43% | 7.39% |
| Independent Independent | 3.60% | 10.30% | 11.69% | 13.74% | 13.51% |
| Independent Democrat | 3.40% | 10.50% | 13.71% | 6.61% | 9.73% |
| Weak Democrat | 15.60% | 20.00% | 19.35% | 13.57% | 22.70% |
| Strong Democrat | 46.00% | 25.94% | 35.28% | 23.13% | 19.64% |
| No response | 0.00% | 0.20% | 0.00% | 0.00% | 8.29% |
| *Party Affiliation* |  |  |  |  |  |
| Republican Party | 28.80% | 32.87% | 19.35% | 46.09% | 22.88% |
| Libertarian Party | 2.60% | 6.34% | 6.45% | 2.78% | 7.57% |
| Democratic Party | 67.60% | 56.24% | 62.90% | 47.83% | 55.14% |
| Green Party | 1.00% | 3.56% | 11.09 | 3.30% | 5.95% |
| No response | 0.00% | 0.99% | 0.20% | 0.00% | 8.47% |

*Note*. Table 1 includes mean and standard deviation for age by platform. For all other demographic measures, we report the percentage of participants who selected each response by platform. Participants could select more than one option for ethnicity. Some percentages may not add to 100% due to rounding.

Participants were paid $0.96 on MTurk, CloudResearch, and Prolific. The payment rate was based on the US federal minimum wage ($7.50 per hour). Payment information was not available from Qualtrics because Qualtrics does not allow researchers to determine participants’ compensation. Note that the aforementioned dollar amounts refer to the compensation that participants actually received. These amounts are different from the costs for the researchers because each platform charges additional fees (i.e., a 20% fee on MTurk, a combination of a 20% fee plus additional charges on CloudResearch, a 30% fee on Prolific, and a flat rate based on survey length and additional restrictions on Qualtrics). Participants recruited through SONA received course credit for their participation. For all platforms that permitted the option (i.e., MTurk, CloudResearch, and Prolific), we recruited participants who had completed a minimum of 100 surveys in the past and had an approval rating of 95% or higher as is standard practice for online data collection in the social sciences [20]. It was not possible to include eligibility requirements based on either the number of past surveys taken or approval rate on Qualtrics or SONA. We did not use demographic quotas (i.e., quotas to ensure the same demographic distributions on all platforms) while recruiting participants.

**Platforms**

Participants were recruited through five commonly used data collection platforms, MTurk, CloudResearch, Prolific, Qualtrics, and SONA. See Supplemental Appendix 1 for information about how we determined which platforms to use in our study.

As each platform operates slightly differently, we describe below how the platforms recruit and compensate participants. MTurk recruits participants for a given study from its own pool of potential workers. When a researcher posts a survey (also called a “hit”) it appears on the dashboard of any worker who qualifies for the study. Workers can then opt to take the survey and are compensated with Amazon credit upon completion.

CloudResearch includes multiple options for data collection. We included CloudResearch’s interface for MTurk (the MTurk Toolkit) in the study. The MTurk Toolkit should not be confused with CloudResearch’s other data collection options (i.e., Prime Panels and a managed research option) which we did not examine in our research. The MTurk Toolkit posts surveys to MTurk through CloudResearch’s interface and applies various filters designed to only include respondents who will provide high-quality data. The data quality filters used in our study included CloudResearch’s approved participants list, a duplicate IP address block, a suspicious geo location block, and a worker country location verification. Participants recruited through CloudResearch’s MTurk Toolkit are paid with Amazon credit.

Prolific is designed similarly to MTurk and uses its own pool of participants. Prolific’s participants are paid cash for completing surveys.

Qualtrics uses a variety of recruitment methods including directly emailing participants. Researchers connect with a representative from Qualtrics who then posts the survey on the researcher’s behalf (for example, in our study we requested that Qualtrics recruit 500 adults from the US). Qualtrics then distributes the survey on other data collection platforms, however which platforms is not specified to the researcher. Participants recruited through Qualtrics are compensated with various rewards including cash, gift cards, air miles, and coupons for food. These rewards are determined based on the participant’s preference.

Our undergraduate sample was recruited through SONA. SONA is a platform where organizations (in our study the University of Wisconsin–Madison Psychology Department) can create their own pool of participants and can be designed to meet the needs of a department or lab. Our SONA participant pool included students who sign up for studies in exchange for course credit.

Participants on MTurk and CloudResearch were prevented from taking the survey on both platforms. It was not possible to implement similar preventions between other platforms because only MTurk and CloudResearch use the same worker IDs.

**Materials**

Our study consisted of an online survey administered through Qualtrics’ survey design software.

**Attention Checks**

We measured attention to the survey through five attention checks. In the first attention check we asked participants to identify the color that was mentioned in the consent form, a measure previously used by Douglas et al. [40]. In the second attention check participants were asked to leave a text-entry box empty. The third attention check occurred as part of the personality inventory to which we added an item asking participants to select “strongly agree” from five potential response options. In the fourth attention check participants reported if they have any type of colorblindness. This colorblindness check was adapted from Kan and Drummey [18]. The various response options included “red-blue colorblindness,” which does not exist. Selecting this option indicated that participants were not paying attention to the question. In the fifth attention check we asked participants to complete a simple arithmetic problem (what is 3 x 4?).

**Personality Inventory**

Participants’ personality traits were assessed using the 50-item version of Costa and McCrae's [37] NEO-PI-R Domains obtained from the International Personality Item Pool (IPIP) [38]. Participants were asked to evaluate the extent to which they agreed with various statements about themselves on a 5-point Likert response scale ranging from “Strongly disagree” to “Strongly agree.” Sample items are “I often feel blue” (neuroticism), “I feel comfortable around people” (extroversion), “I have a vivid imagination” (openness), “I believe that others have good intentions” (agreeableness) and “I pay attention to details” (conscientiousness).

**Sustainability**

Participants were shown one of three 75-second videos about sustainability mid-way through the survey. Participants were later shown short descriptions of each of the three videos and were asked to choose the description of the video that matched what they had seen. Correct identification of the description indicated correct recall of the video. Participants were asked a series of follow-up questions including the extent to which they thought the government should invest in green energy. Participants were asked this same item again 21 questions later in the survey to assess the test-retest correlation of participant responses.

**Belief in Conspiracy Theories**

We measured participants’ belief in conspiracy theories using Brotherton et al.’s [41] Belief in Conspiracy Theories Scale. The scale includes 15 items each describing a different conspiracy theory (e.g., “Some UFO sightings and rumors are planned or staged in order to distract the public from real alien contact”). Participants responded to each of the items on a 5-point Likert scale ranging from “Definitely not true” to “Definitely true.”

**Experience Taking Online Surveys**

Participants were asked the frequency with which they take online surveys. We asked participants how many surveys they think they had taken over the past seven days and over the past year.

**Survey Meta-data**

We also measured the amount of time each participant spent completing the survey. We categorized participants who took the survey in more than 3 minutes as having passed the speed indicator. The specific 3-minute cutoff was determined by having a research assistant take the survey as fast as possible. Three minutes was the fastest the survey could be completed while still reading each question. Additionally, because participants completed 106 survey items and watched one of three approximately 75-second videos, if participants spent 1 second per question the survey should take (at a minimum) 181 seconds to complete. Thus, the 3-minute cutoff was also consistent with Wood et al.’s [33] finding that response quality drops if participant spend only 1 second on each question. We also recorded participants’ IP address, geolocation, worker ID, and the number of missing responses throughout the survey. Having a unique worker ID, IP address, and geolocation were also used to indicate high-quality data as these identifiers should be unique to each participant.

**Demographics**

We included demographic measures for age, gender identity, identity as transgender, ethnicity, sexual orientation, family income, highest level of education completed, political affiliation, party affiliation, and political opinions. To measure political affiliation, we used the American National Election Studies’ (ANES) Party Identification 7-Point Scale [42]. This measure involves a two-step procedure in which participants first are asked if they identify as a Democrat, Republican, or neither. If participants identify as a Democrat or Republican, they are then asked to specify how strongly they identify with that party. If participants identify as neither, they are prompted to select if they feel closer to the Democratic Party, Republican Party, or neither. Participants also reported their party affiliation from one of four political parties they would vote for in a hypothetical state election (Republican Party, Libertarian Party, Democratic Party, and Green Party). We included measures of political opinion with respect to social and economic issues (e.g., “Please select the statement that most closely describes your political beliefs on social issues” with 5 response options ranging from “I am very conservative” to “I am very liberal”).

**End of Survey Questions**

Participants were given the opportunity to provide feedback in an open-response text box. We also asked participants to check a box if they believed their data should be considered low-quality. Participants were told that responding to the survey while “distracted, respond[ing] to questions without reading them, or pick[ing] answers randomly” could lead to low-quality data. Selecting the box to indicate low-quality data did not affect their payment.

**Procedure**

Prior to posting the survey on each of the platforms we reached out to representatives from CloudResearch, Prolific, and Qualtrics to ensure that we were using all possible means to guarantee high data quality. It was not possible to reach out to MTurk as they do not have a representative we could contact. Prior to posting our study on SONA, the study was reviewed by our department’s Introduction to Psychology Research Coordinator who oversees the department’s SONA participant pool. We did not receive funding from any of the platforms. The survey was estimated to take 8 minutes to complete.

**Ethics Statement**

This study was approved by the Minimal Risk Research Board of the University of Wisconsin–Madison Institutional Review Board. A waiver of signed consent was approved by the IRB. Participants were required to read the consent form, agree to participate, but not sign the form prior to participating in the study.

**Results**

We conducted 11 inferential tests per outcome measure, 10 tests that compared each platform to each of the other platforms and 1 overall 4-df test examining the null hypothesis that there were no differences between the five platforms. For continuous outcomes we used regular regression and conducted standard (Fisher) *F*-tests. For dichotomous outcomes we used logistic regression and conducted likelihood ratio tests that yielded a Chi-square value. In all analyses we used dummy codes to do pairwise comparisons between platforms. We created a set of four dummy codes with MTurk as the reference group and tested their statistical significance without any kind of adjustment. Given we had no a priori hypotheses about differences in data quality between the other four platforms, we did post-hoc pairwise comparisons between Prolific, CloudResearch, Qualtrics, and SONA using different sets of dummy codes and applying a Holm-Bonferroni adjustment to determine statistical significance (i.e., to account for the fact that we already “used up” all available degrees of freedom for the test of our a priori hypotheses) [43].

Participants from SONA were not included in the analysis for the unique IP address and unique geolocation outcome measures as these participants were all located on the same university campus and thus these participants should share IP addresses and geo locations. For these two sets of analyses, we conducted a total of 7 inferential tests, 6 tests that compared each platform to each of the other platforms and 1 overall 3-df test examining the null hypothesis that there were no group differences.

Additionally, because device type (mobile device vs. desktop/laptop computer) affects the presentation of the survey, we controlled for device type when regressing completion time (a continuous outcome measure) on each of the four sets of dummy codes.

To assess the test-retest reliability, we examined the strength of the relationship between participants’ response to the item “the government should invest in green energy” and their response to the same item presented later in the survey. More precisely, we regressed participants’ time 2 response on their time 1 response, 4 dummy codes, and the 4 interaction terms involving the time 1 response and each of the dummy codes. By running several regression analyses with different sets of dummy codes, we were able to examine if the test-retest reliability of each platform was statistically different from that of each of the other platforms. We again applied no adjustment for the a priori comparisons involving MTurk, but a Holm-Bonferroni adjustment for the post-hoc comparisons not involving MTurk.

Results for various dichotomous indicators of data quality are presented in Table 2. In general, participants provided higher quality data on Prolific and CloudResearch than on MTurk, Qualtrics, or SONA. We found significant differences between platforms for all of the dichotomous outcomes: the percentage of respondents who selected the response option “Strongly agree” when instructed to do so, passed a simple arithmetic attention check, gave a meaningful answer to the question about recalling the color mentioned in the consent form, left the textbox blank when instructed to do so, answered the question about colorblindness in a meaningful manner, had a unique worker ID, had a unique IP address, had a unique geolocation, completed the survey in more than 3 minutes, gave a meaningful or blank response when asked an open-ended question, and reported at the end of the survey that their data were of high quality and should be included in the data analyses.

**Table 2. Percent of Participants with High-Quality Data for the Dichotomous Outcome Measures, and the Cost We Paid per High-Quality Respondent Broken Down by Platform. "High-Quality Respondents" are Those Who Passed 4 or More Attention Checks (rows 1-5) and the Other Criteria Reported (rows 6-11).**

| **Outcome Measure** | **MTurk**  **(N = 500)** | **CloudResearch**  **(N = 505)** | **Prolific**  **(N = 496)** | **Qualtrics**  **(N = 575)** | **SONA**  **(N = 555)** |
| --- | --- | --- | --- | --- | --- |
| Select Strongly Agree* | 93.80%^a^_vw_ | 97.03%^b^_vx_ | 98.39%^b^_x_ | 93.04%^a^_wy_ | 89.01%^c^_vy_ |
| Pass Arithmetic Check* | 98.40%^a^_v_ | 99.41%^a^_v_ | 99.19%^a^_v_ | 97.74%^a^_v_ | 91.71%^b^_v_ |
| Pass Color Recall Check* | 75.76%^a^_v_ | 95.84%^b^_w_ | 98.58%^c^_x_ | 93.56%^b^ | 92.80%^b^_y_ |
| Leave Textbox Blank* | 98.80%^a^_v_ | 100.00%^b^_w_ | 100.00%^b^_w_ | 99.83%^b^_vw_ | 99.64%^ab^_vw_ |
| Pass Colorblindness Check* | 88.60%^a^_v_ | 98.02%^b^_w_ | 98.59%^b^_w_ | 93.90%^c^_vx_ | 98.59%^b^_wx_ |
| Unique Worker ID* | 99.60%^a^_v_ | 100.00%^a^_v_ | 100.00%^a^_v_ | 100.00%^a^_v_ | 77.12%^b^_w_ |
| Unique IP Address* | 97.20%^a^_v_ | 99.21%^b^_w_ | 98.79%^a^_vw_ | 100.00%^c^_w_ | - |
| Unique Geolocation* | 53.40%^a^_v_ | 90.10%^b^_w_ | 89.92%^b^_w_ | 88.70%^b^_w_ | - |
| Time > 3 Minutes* | 82.60%^a^_v_ | 88.91%^b^_w_ | 90.12%^b^_w_ | 78.96%^a^_v_ | 81.98^a^_v_ |
| Meaningful or Blank Open Response* | 82.20%^a^_v_ | 99.01%^b^_w_ | 99.19%^b^_w_ | 99.30%^b^_w_ | 99.64%^b^_w_ |
| Self-Reported High Data Quality* | 54.40%^a^_v_ | 79.80%^b^_w_ | 85.89%^c^_x_ | 85.39%^c^_w_ | 77.12%^b^_w_ |
| High-Quality Respondents* | 26.40%^a^_v_ | 61.98%^b^_wx_ | 67.94%^c^_x_ | 53.22%^d^_y_ | 52.79%^d^_wy_ |
| Total Cost | $575 | $625 | $640 | $2500 | $0 |
| Cost We Paid per High-Quality Respondent | $4.36 | $2.00 | $1.90 | $8.17 | $0.00 |

*Note*. Higher percentages indicate higher data quality on each outcome measure. Percentages with different superscripts a-e are significantly different at the *p* < .05 threshold. A * indicates that the overall multiple-df test was statistically significant at the *p* < .05 threshold. Percentages with different subscripts v-y are significantly different at the *p* < .05 threshold when controlling for participants’ age, gender, ethnicity, sexual orientation, income, and level of education. Measures used to compute the composite High-Quality Respondents score (line 12) are included in lines 1 – 11 of Table 2.

We computed the percentage of high-quality respondents for each platform. A respondent was classified as high-quality when they failed no more than 1 of the five attention checks, had a unique worker ID, IP address, and geolocation, completed the survey in 3 minutes or longer, provided meaningful responses to (or left blank) the optional open-response text box, and indicated that their data were high quality. Prolific and CloudResearch – and to a lesser extent Qualtrics and SONA – had a considerably greater percentage of high-quality respondents than MTurk (see Table 2). We further computed how much money we spent per high-quality respondent. With $1.90 and $2.00 Prolific and CloudResearch were “the best deals,” whereas MTurk ($4.36) and Qualtrics ($8.17) had a worse quality-price ratio among paid platforms. Since SONA participants were not monetarily compensated, the cost was $0.00. While recruitment on the four paid platforms took a matter of days, recruitment on SONA took the duration of a semester (14 weeks).

Fewer participants had unique worker IDs on SONA than any other platform. The low percentage of unique worker IDs was attributed to participants opening the survey via SONA, then terminating the survey before viewing all of the survey items, and then re-taking the survey. These partial responses on SONA also help explain why SONA had more missing responses to survey items than any of the other platforms (see Table 3).

**Table 3. Descriptive Statistics for the Continuous Outcome Measures Directly or Indirectly Related to Data Quality, Broken Down by Platform**

| **Measure** | **MTurk**  **(N = 500)** | **CloudResearch**  **(N = 505)** | **Prolific**  **(N = 496)** | **Qualtrics**  **(N = 575)** | **SONA**  **(N = 555)** |
| --- | --- | --- | --- | --- | --- |
| Number of Missing Responses* | 0.03^a^_v_ (0.18) | 0.03^a^_v_ (0.24) | 0.02^a^_v_ (0.26) | 0.01^a^_v_ (0.16) | 6.34^b^_v_ (22.51) |
| Personality Inventory (mean scores) |  |  |  |  |  |
| Neuroticism* | 2.66^a^_v_ (0.67) | 2.37^b^_w_ (0.95) | 2.79^c^_v_ (0.94) | 2.35^b^_v_ (0.77) | 2.74^ac^_w_ (0.74) |
| Extroversion* | 3.14^a^_v_ (0.61) | 3.02^b^_w_ (0.85) | 2.90^c^_w_ (0.89) | 3.04^b^_w_ (0.72) | 3.48^d^_x_ (0.72) |
| Openness* | 3.36^a^_v_ (0.61) | 3.74^b^_w_ (0.75) | 3.92^c^_x_ (0.68) | 3.35^a^_v_ (0.67) | 3.70^b^_wx_ (0.59) |
| Agreeableness* | 3.43^a^_v_ (0.64) | 3.92^b^_w_ (0.64) | 3.75^c^_x_ (0.62) | 3.95^b^_x_ (0.57) | 3.87^b^_w_ (0.52) |
| Conscientiousness* | 3.50^a^_v_ (0.67) | 3.98^b^_w_ (0.70) | 3.63^c^_x_ (0.77) | 3.91^b^_vx_ (0.63) | 3.60^c^_x_ (0.64) |
| Personality Inventory (Cronbach’s Alphas) |  |  |  |  |  |
| Neuroticism (IPIP = .86) | .70 | .92 | .90 | .88 | .85 |
| Extraversion (IPIP = .86) | .67 | .89 | .89 | .86 | .87 |
| Openness (IPIP = .82) | .65 | .82 | .79 | .78 | .74 |
| Agreeableness (IPIP = .77) | .72 | .83 | .79 | .82 | .75 |
| Conscientiousness (IPIP = .81) | .75 | .88 | .88 | .86 | .82 |
| Belief in Conspiracy Theories* | 3.26^a^_v_ (1.02) | 2.46^bc^_wx_ (1.04) | 2.61^b^_w_ (0.97) | 2.43^c^_w_ (0.95) | 2.56^b^_x_ (0.85) |
| Correlation Between T1 and T2 Response to the Same Question* | .54^a^_v_ | .87^b^_w_ | .87^b^_w_ | .88^b^_w_ | .69^c^_x_ |
| Number of Surveys Taken (7 Days)* | 45.99^a^_v_  (96.79)  [15.00] | 59.05^a^_v_ (70.64) [40.00] | 19.15^ab^_vw_ (22.01) [13.50] | 37.39^ab^_vw_ (604.64) [5.00] | 1.97^b^_w_ (4.63) [2.00] |
| Number of Surveys Taken (1 Year)* | 1554.95^a^_v_ (5827.10) [130.00] | 1735.53^a^_v_ (3306.19) [800.00] | 598.38^b^_w_ (3602.91) [170.00] | 300.76^bc^_x_ (1012.11) [50.00] | 6.68^c^_x_ (30.26) [3.00] |
| Completion Time (Seconds)* | 746.07^a^_v_ (450.40) [618.50] | 647.54^a^_v_ (311.40) [568.00] | 692.81^a^_v_ (398.07) [625.00] | 1017.27^a^_v_ (1262.26) [762.00] | 6389.89^b^_v_ (42804.54) [715.00] |

*Note*. The reported values are mean values for each platform. Standard deviations are reported in parentheses. For highly skewed variables we also present median values in brackets. Means with different superscripts a-d are significantly different at the *p* < .05 threshold. A * indicates that the overall multiple-df test was statistically significant. Means with different subscripts v-x are significantly different at the *p* < .05 threshold when controlling for participants’ age, gender, ethnicity, sexual orientation, income, and level of education. The IPIP values represent Cronbach’s Alphas values reported by the International Personality Inventory Pool and come from an existing sample of *N* = 856 participants [37]. For MTurk, CloudResearch, and Prolific, participants were only eligible to take our survey if they had previously completed a minimum of 100 surveys.

Participants reported their recall of the content from a video about sustainability. A significantly smaller proportion of participants correctly recalled the content of the video on MTurk (52.20%) than on any of the other platforms. Among the other platforms, a greater percentage of participants on CloudResearch (81.78%) and Prolific (83.47%) correctly recalled the video content than on Qualtrics (74.96%) and SONA (72.99%). There were no significant differences in the percentage of participants who correctly recalled the video between CloudResearch and Prolific nor between Qualtrics and SONA.

We also examined several continuous outcome measures that were directly or indirectly related to data quality (see Table 3). Only SONA had significantly more missing responses than any other platform. MTurk, Prolific, and SONA respondents scored higher on neuroticism and lower on conscientiousness in the Personality Inventory than respondents from CloudResearch and Qualtrics. In general, the scale reliabilities for the MTurk sample were lower than those of both the other platforms and a normed reference group (IPIP) [36]. MTurk participants were more likely to believe in conspiracy theories than participants from any of the other four platforms. The relationship between participants’ opinion about green energy investment and that same opinion measured later in the survey was considerably smaller on MTurk than on the other four platforms. MTurk and CloudResearch participants reported having taken more surveys in the last year than Prolific participants. Qualtrics participants had taken more surveys than SONA participants who reported the fewest number of surveys taken. Additional findings concerning how representative each platform was of the US population and how our results change after the removal of low-quality respondents can be found in Supplemental Appendix 1.

**Discussion**

We sought to determine which online data collection platforms produced the highest quality data. We compared MTurk, CloudResearch, Prolific, Qualtrics, and an undergraduate student sample (i.e., SONA). CloudResearch and Prolific provided higher quality data than MTurk, Qualtrics, and SONA. The cost we paid per high-quality respondent was approximately $2.00 for CloudResearch and Prolific, the cost was more than twice that on MTurk and over four times the cost on Qualtrics. Collecting participants on SONA was free, thus the cost we paid per high-quality respondent was the lowest for SONA. However, researchers may consider factors in addition to cost, such as the time required to collect responses, when making decisions about which platform is ideal for a given study. Overall, Prolific and CloudResearch provided the highest quality data, for the lowest price.

Our results expand upon previous findings showing that both Prolific and CloudResearch provide high-quality data [e.g., 7] by providing a cost estimate per high-quality respondent for each platform. The cost estimate also offers a starting point when considering other platforms for data collection. For example, previous researchers examining data quality on Dynata describe paying $2.50 per completed survey yet found worse data quality than provided by MTurk [44]. While it is not possible for us to estimate a cost per participant on a platform we did not examine, one could predict that a platform with previously identified low-quality data and high costs would be less cost efficient than other options with high-quality data and low costs. Our cost estimates also provide a comparison point with other approaches to online data collection. All of the platforms we examined cost less per high-quality participant than previously reported costs per participant (regardless of quality) for online advertisements on websites like Facebook [45]. It should be noted however, that the cost of data collection on each of the platforms depends on how one measures data quality. For example, were one to remove the self-reported data quality measure from our results the (relatively minor) difference in cost between CloudResearch and Prolific would diminish. Additionally, because previous findings have demonstrated that increasing participant pay can improve data quality [16], these cost estimates may differ with different pay rates. Overall, our findings regarding the cost we paid per high-quality respondent provide an initial comparison point for researchers considering which data collection platform is best suited to a given project (and that project’s budget).

Several of the outcome measures included in our study speak to data quality indirectly and should be considered as an indicator of data quality on a case-by-case basis. For example, we included Brotherton et al.’s [41] Belief in Conspiracy Theories Scale. That MTurk participants had significantly higher average scores on this scale does not inherently mean that MTurk produces worse data quality. However, if we imagine a team of researchers studying the public’s trust in the government, MTurk may or may not be the ideal platform for their study. Similar conclusions can be drawn about participant’s responses to the personality inventory. While there is nothing inherently better or worse about having more neurotic or conscientious individuals take a survey, researchers studying personality should consider which participant pool will best allow them to test their research question.

Our results support previous findings about “professional” survey takers (individuals who take hundreds or thousands of surveys per year). For example, Eisele et al., [46] observed that frequently surveyed individuals do not provide worse quality data than less frequently surveyed individuals. Likewise, Peer et al. [19] observed that MTurk (the platform with the most experienced workers) had worse data quality than Prolific (one of two platforms with less experienced workers) but better data quality than CrowdFlower (the other platform with less experienced workers). These latter findings would suggest that experience taking surveys alone does not account for differences in data quality. We found the same pattern of results in our study. CloudResearch participants, who completed the most surveys per year, had among the highest quality data. Meanwhile MTurk, Qualtrics, and SONA participants provided among the lowest quality data yet MTurk participants were highly experienced while participants on Qualtrics and SONA were not. It is possible that our decision to seek out participants who completed a minimum of 100 surveys could have hindered our ability to determine if a relationship between experience and data quality existed. However, given that such requirements are standard practice for online data collection [20] and the previous findings showing no direct relationship between experience and data quality, it seems unlikely that survey experience explains why platforms differ with respect to data quality. Future studies examining this particular relationship would be advised to examine how including (or not including) the 100-survey minimum requirement affects data quality.

Subsequent research may also examine how time-based cutoffs affect the assessment of data quality. While our 1-second-per-item cutoff is supported by past research [33], it is not necessarily the case that a participant who passed this data quality check truly attended to each question. For example, the items on the Belief in Conspiracy Theories Scale [41] take more than a single second to read, much less fully comprehend. Even if someone were able to read the items in such a short amount of time, it is unlikely they thoughtfully considered the implications of each statement long enough to accurately report their agreement with the items. As such, while failing the 1-second cutoff indicates an individual did not read all questions carefully, passing the cutoff is not sufficient on its own to determine that someone has carefully responded to each question.

Our cost per high-quality respondent result is unique among studies assessing online data quality. However, it is by no means the final say over which platforms will always provide the best value per respondent. Take, for example, our decision to include only individuals who had previously completed 100 or more surveys. While such a restriction can be implemented on MTurk, CloudResearch, and Prolific, this restriction is not possible on Qualtrics. As such, the Qualtrics sample may more accurately reflect data quality among novice participants than the other paid platforms. Thus, if one were to imagine a researcher interested in recruiting a novice sample, it is conceivable that Qualtrics could be a more competitive option against other platforms like CloudResearch or Prolific in terms of cost per high-quality respondent. Likewise, researchers may be interested in using quotas to guarantee they obtain a sample which reflects the demographics of the general population. Our Qualtrics sample had a mean age of 64, making it the platform with an average age furthest from the US national average age (38 years old). Would the observed differences in data quality disappear had we used quotas to ensure the mean age on all platforms match the US population? While our results do not change when controlling for the demographic characteristics of the samples, we cannot directly answer this question. Our conclusions about data quality are most applicable to researchers using the same recruitment criteria that we used. Likewise, in the present study we examined only five popular data collection platforms. Future studies should investigate a broader selection of platforms to determine if there is a better option for online data collection than those included in our study. In sum, our results provide a starting point for researchers considering where to launch online surveys, but researchers should consider the advantages and disadvantages to all platforms prior to collecting their data.

Our results should be replicated in future studies at regular intervals. As observed by Arechar and Rand [22], online research updates frequently and can be affected by events external to the platforms. Even in the short time since collecting the data presented in this article, a viral video prompted many young White women to join Prolific’s participant pool. As such the demographic composition from our Prolific sample may differ from what researchers find in a year from now. Relatedly, while we provide a comparison between the demographic results observed in our study and those of the general population in Supplemental Appendix 1, multiple studies would be needed to definitively determine how representative a particular platform is of the US population. Overall, Prolific and CloudResearch provided the highest quality data for the lowest cost.

**References**

1. Buhrmester MD, Talaifar S, Gosling SD. An evaluation of Amazon’s Mechanical Turk, its rapid rise, and its effective use. Perspect Psychol Sci. 2018; 13: 149-54.
2. Chmielewski M, Kucker SC. An MTurk crisis? Shifts in data quality and the impact on study results. Soc Psychol Personal Sci. 2020; 11: 464-73.
3. Huang JL, Liu M, Bowling NA. Insufficient effort responding: examining an insidious confound in survey data. J Appl Psychol. 2015; 100: 828.
4. Huang JL, DeSimone JA. Insufficient effort responding as a potential confound between survey measures and objective tests. J Bus and Psychol. 2021; 36: 807-828.
5. Chandler J, Sisso I, Shapiro D. Participant carelessness and fraud: Consequences for clinical research and potential solutions. J Abnorm Psychol. 2020;129: 49-55.
6. Barends AJ, de Vries RE. Noncompliant responding: Comparing exclusion criteria in MTurk personality research to improve data quality. Pers Individ Dif. 2019; 143: 84-9.
7. Eyal P, David R, Andrew G, Zak E, Ekaterina D. Data quality of platforms and panels for online behavioral research. Bev Res Methods. 2021;1-20.
8. MacInnis B, Krosnick JA, Ho AS, Cho MJ. The accuracy of measurements with probability and nonprobability survey samples: Replication and extension. Public Opin Q. 2018; 82: 707-44.
9. Yeager DS, Krosnick JA, Chang L, Javitz HS, Levendusky MS, Simpser A, Wang R. Comparing the accuracy of RDD telephone surveys and internet surveys conducted with probability and non-probability samples. Public Opin Q. 2011; 75: 709-47.
10. Cornesse C, Blom AG, Dutwin D, Krosnick JA, De Leeuw ED, Legleye S, Pasek J, Pennay D, Phillips B, Sakshaug JW, Struminskaya B. A review of conceptual approaches and empirical evidence on probability and nonprobability sample survey research. Journal of Survey Statistics and Methodology. 2020; 8: 4-36.
11. Kennedy C, Hatley N, Lau A, Mercer A, Keeter S, Ferno J, Asare-Marfo D. Assessing the Risks to Online Polls from Bogus Respondents. [Internet]. Pew Research Center; 2020. Available from: https://www.pewresearch.org/methods/2020/02/18/assessing-the-risks-to-online-polls-from-bogus-respondents/
12. Buhrmester MD, Kwang T, Gosling SD. Amazon’s Mechanical Turk: A new source of inexpensive, yet high-quality, data? Perspect Psychol Sci. 2011; 6: 3–5.
13. Roulin N. Don't throw the baby out with the bathwater: Comparing data quality of crowdsourcing, online panels, and student samples. Ind Organ Psychol. 2015; 8: 190-6.
14. Kees J, Berry C, Burton S, Sheehan K. An analysis of data quality: Professional panels, student subject pools, and Amazon's Mechanical Turk. J Advert. 2017; 46: 141-55.
15. Smith SM, Roster CA, Golden LL, Albaum GS. A multi-group analysis of online survey respondent data quality: Comparing a regular USA consumer panel to MTurk samples. J Bus Res. 2016; 69: 3139-48.
16. Litman L, Robinson J, Rosenzweig C. The relationship between motivation, monetary compensation, and data quality among US-and India-based workers on Mechanical Turk. Behav Res Methods. 2015; 47: 519-28.
17. Necka EA, Cacioppo S, Norman GJ, Cacioppo JT. Measuring the prevalence of problematic respondent behaviors among MTurk, campus, and community participants. PloS one. 2016; 11: e0157732.
18. Kan IP, Drummey AB. Do imposters threaten data quality? An examination of worker misrepresentation and downstream consequences in Amazon's Mechanical Turk workforce. Comput Human Behav. 2018; 83: 243-53.
19. Peer E, Brandimarte L, Samat S, Acquisti A. Beyond the Turk: Alternative platforms for crowdsourcing behavioral research. J Exp Soc Psychol. 2017; 70: 153-63.
20. Chandler J, Rosenzweig C, Moss AJ, Robinson J, Litman L. Online panels in social science research: Expanding sampling methods beyond Mechanical Turk. Behav Res Methods. 2019; 51: 2022-38.
21. Ibarra JL, Agas JM, Lee M, Pan JL, Buttenheim AM. Comparison of online survey recruitment platforms for hard-to-reach pregnant smoking populations: Feasibility study. JMIR Res Protoc. 2018; 7: e101.
22. Arechar AA, Rand DG. Turking in the time of COVID. Behav Res Methods. 2021; 53: 2591-2595.
23. Ternovski J, Orr L. A Note on Increases in Inattentive Online Survey-Takers Since 2020. Journal of Quantitative Description: Digital Media. 2022; 2: 1-35.
24. Coppock A, McClellan OA. Validating the demographic, political, psychological, and experimental results obtained from a new source of online survey respondents. Res Politics. 2019; 6: 1-14.
25. Litman L, Moss A, Rosenzweig C, Robinson J. Reply to MTurk, Prolific or panels? Choosing the right audience for online research. Choosing the right audience for online research. 2021.
26. Bowling NA, Gibson AM, Houpt JW, Brower CK. Will the questions ever end? Person-level increases in careless responding during questionnaire completion. Organ Res Methods. 2021; 24: 718-38.
27. Hauser DJ, Sunderrajan A, Natarajan M, Schwarz N. Prior exposure to instructional manipulation checks does not attenuate survey context effects driven by satisficing or gricean norms. MDA. 2016; 10: 195-220.
28. Hauser DJ, Ellsworth PC, Gonzalez R. Are manipulation checks necessary?. Front Psychol. 2018; 9: 998.
29. Curran PG, Hauser KA. I’m paid biweekly, just not by leprechauns: Evaluating valid-but-incorrect response rates to attention check items. J Res Pers. 2019; 82: 103849.
30. Wise SL, Kong X. Response time effort: A new measure of examinee motivation in computer-based tests. Appl Meas Educ. 2005; 18: 163-83.
31. Malhotra N. Completion time and response order effects in web surveys. Public Opin Q. 2008; 72: 914-934.
32. Zhang C, Conrad F. Speeding in web surveys: The tendency to answer very fast and its association with straightlining. Surv Res Methods. 2014; 8: 127-135.
33. Wood D, Harms PD, Lowman GH, DeSimone JA. Response speed and response consistency as mutually validating indicators of data quality in online samples. Soc Psychol Personal Sci. 2017; 8: 454-64.
34. Aguinis H, Villamor I, Ramani RS. MTurk research: Review and recommendations. J Manag. 202; 47: 823-37.
35. Rouse SV. A reliability analysis of Mechanical Turk data. Comput Human Behav 2015; 43: 304-7.
36. Goldberg LR. A broad-bandwidth, public domain, personality inventory measuring the lower-level facets of several five-factor models. Personality psychology in Europe. 1999; 7: 7-28.
37. Costa PT Jr, McCrae RR. (1992). Revised NEO Personality Inventory (NEO PI-R™) and NEO Five-Factor Inventory (NEO-FFI): Professional manual. Odessa, FL: Psychological Assessment Resources.
38. Goldberg LR, Johnson JA, Eber HW, Hogan R, Ashton MC, Cloninger CR, Gough HG. The international personality item pool and the future of public-domain personality measures. J Res Pers. 2006; 40: 84-96.
39. Huang JL, Curran PG, Keeny J, Poposki EM, DeShon RP. Detecting and deterring insufficient effort responding to surveys. J Bus Psychol. 2012; 27: 99-114
40. Douglas BD, McGorray EL, Ewell PJ. Some researchers wear yellow pants, but even fewer participants read consent forms: Exploring and improving consent form reading in human subjects research. Psychol Methods. 2021; 26: 61.
41. Brotherton R, French CC, Pickering AD. Measuring belief in conspiracy theories: The generic conspiracist beliefs scale. Front Psychol. 2013; 279.
42. The American National Election Studies (ANES). 2020. Party Identification 7-Point Scale 1952-2020 [dataset]. Available from: https://electionstudies.org/resources/anes-guide/top-tables/?id=21
43. Holm S. A simple sequentially rejective multiple test procedure. Scand Stat Theory Appl. 1979; 65-70.
44. Zhang B, Gearhart S. Collecting online survey data: A comparison of data quality among a commercial panel & MTurk. Surv. Pract. 2020; 13: 1-10.
45. Antoun C, Zhang C, Conrad FG, Schober MF. Comparisons of online recruitment strategies for convenience samples: Craigslist, Google AdWords, Facebook, and Amazon Mechanical Turk. Field Methods. 2016; 28: 231-46.
46. Eisele G, Vachon H, Lafit G, Kuppens P, Houben M, Myin-Germeys I, Viechtbauer W. The effects of sampling frequency and questionnaire length on perceived burden, compliance, and careless responding in experience sampling data in a student population. ASMNT. 2022; 29:136-51.

**Supporting Information**

**SA1**. This is Supporting Appendix 1.

1. Some researchers may also be interested in data quality from the perspective of external validity. While representativeness is not the primary concern of this article, we provide a comparison between the demographic characteristics of the participants from each platform’s sample and the US population in Supplemental Appendix 1. [↑](#footnote-ref-1)
